# Transabdominal Preperitoneal Repair With Concomitant Bowel Resection for Strangulated Femoral Hernias: A Report of Five Cases

**DOI:** 10.7759/cureus.109460

**Published:** 2026-05-22

**Authors:** Tatsuya Suzuki, Yasuhiro Yamamoto, Kengo Shigehara, Daisei Watanabe, Katsuro Enomoto, Takahiro Nishikoshi

**Affiliations:** 1 Department of Surgery, Rumoi City Hospital, Rumoi, JPN; 2 Department of Surgery, Kutchan-Kosei General Hospital, Kutchan, JPN; 3 Department of Surgery, Honjo-General Hospital, Honjo, JPN; 4 Department of Surgery, Kobayashi Hospital, Kitami, JPN

**Keywords:** bowel resection, small intestinal resection, strangulated femoral hernia, strangulated groin hernia, tapp, transabdominal preperitoneal repair

## Abstract

It is controversial whether prosthetic mesh can be used laparoscopically in emergency groin hernia repair when bowel resection is needed. While the guideline for emergency hernia repair recommends the use of prosthetic mesh in a clean-contaminated wound based on the studies with the open anterior approach, evidence supporting the laparoscopic use of prosthetic mesh in a clean-contaminated wound is scarce. The present paper reports the procedure and the clinical outcome of five patients treated by transabdominal preperitoneal (TAPP) repair with concomitant bowel resection for strangulated femoral hernia between 2015 and 2024 at Kobayashi Hospital, Kitami, Japan. The hernia was laparoscopically reduced by enlargement of the hernia orifice with traction and compression on the intestine, followed by TAPP repair and extracorporeal small intestinal resection at the same surgery. There was no conversion to open surgery or infectious complications. The results of our report and earlier literature support that TAPP repair with concomitant bowel resection for strangulated groin hernia appears to be feasible and safe, although further studies are needed to verify the safety of this procedure.

## Introduction

Laparoscopic approaches for groin hernia repair are widely performed in elective surgery and have advantages such as faster recovery and less chronic pain compared to open anterior approaches [1]. In light of these benefits, surgeons performing a laparoscopic hernia repair in usual practice may prefer laparoscopic approaches in an emergency setting as well as in an elective setting.

Evidence is accruing that laparoscopic groin hernia repairs are feasible and safe even in emergency surgery [2,3]. Given that laparoscopic approaches for acute groin hernias have advantages of shorter hospital stays and less postoperative morbidity over conventional open approaches, it may bring benefits to patients, especially in the elderly population with high morbidity and mortality rates [4,5]. Some reports included cases of laparoscopic groin hernia repairs with concomitant bowel resection, and the incidence of complications associated with the procedures was preferably low in their reports [6-9].

We have performed laparoscopic groin hernia repairs at either an elective or emergency surgery in our hospital since 2015, and experienced several cases of laparoscopic groin hernia repairs with concomitant bowel resection. However, it still remains controversial whether a prosthetic mesh should be placed in the groin laparoscopically in a clean-contaminated wound where a strangulated bowel is resected at the same surgery. However, the guideline for emergency hernia repair recommends the use of prosthetic mesh in a clean-contaminated wound based on the studies with herniorrhaphy through an open anterior approach [10]. Not many studies have been made on the feasibility and safety of the laparoscopic use of prosthetic mesh in a clean-contaminated wound for a strangulated groin hernia.

The present paper reports the clinical outcome of five cases of strangulated femoral hernias treated by transabdominal preperitoneal (TAPP) repair with concomitant bowel resection to support the feasibility and safety of TAPP with concomitant bowel resection for the treatment of strangulated groin hernias.

## Case presentation

We experienced five cases of strangulated femoral hernias operated on with hernia reduction, laparoscopic mesh herniorrhaphy, and extracorporeal resection of the small intestine due to ischemia at Kobayashi Hospital, Kitami, Japan, between 2015 and 2024.

With regard to patient selection, we routinely used the TAPP approach for strangulated groin hernia as a first choice in this period. We considered mesh placement in the groin where a bowel had been strangulated to be suitable unless there was a sign of infection, such as purulent exudate. Contraindications to the TAPP approach were intestinal perforation with peritonitis, a history of prostatectomy, and severe bowel distension due to extensive bowel obstruction. An extremely large hernia was operated on by the anterior approach. The comorbidities did not affect the patient selection.

Surgery was performed as follows: under general anesthesia, a 12-mm camera port was inserted at the umbilicus. The abdomen was insufflated with carbon dioxide at 10 mmHg. Two 5-mm ports were placed on both sides of the abdomen to allow triangulation. At first, the hernia was reduced laparoscopically in all cases except Case 1. The hernia sac was flushed out with saline in three cases using a 12-F Nelaton catheter, but the hernia was not reduced. Then, a peritoneal incision was made medially towards the lacunar ligament in order to enlarge the hernia orifice using an ultrasonically activated device (SonoSurg®; Olympus, Tokyo, Japan) (Figure 1A). The tissue pad of the SonoSurg® was inserted between the hernia sac and the small intestine to avoid tissue injury by the active blade. The small intestine was reduced by gentle traction with grasping forceps and manual compression from the outside (Figures 1B, 1C). Then, TAPP repair was performed. The parietal peritoneum was incised to expose the preperitoneal layer of the myopectineal orifice. The hernia sac was left in situ in all cases except in Case 4, where it was resected. The diameter of the hernia orifice was measured before mesh placement. A prosthetic mesh (3D Max® Light Mesh, M size, 7.9 x 13.4 cm; Bard, Warwick, Rhode Island, USA) was used for covering the myopectineal orifice and fixed by non-absorbable tacks (CapSure®; Bard), except for Case 5 (Figure 1D). The peritoneum was closed tightly by using a 3-0 absorbable barbed suture (V-Loc®; Covidien, Mansfield, MA, USA) or a 3-0 absorbable braided suture (Vicryl®; Ethicon, Raritan, NJ, USA) in a running fashion (Figure 1E). After the repair was done, the viability of the released bowel was assessed by the laparoscopic inspection of its color; the bowel was judged nonviable and resected if it was dark red or dark purple, whereas it was judged viable and preserved if it was bright red or light red. In all five cases presented, the strangulated small bowel was resected at the same surgery. The extracorporeal resection of the bowel and anastomosis in a functional end-to-end fashion with linear staplers (Endo GIA^TM^ or GIA^TM^; Covidien) was performed through a 3.0- to 5.0-cm umbilical incision (Figure 1F). No drain was inserted. All surgeries were supervised or performed by experienced surgeons.

**Figure 1:**
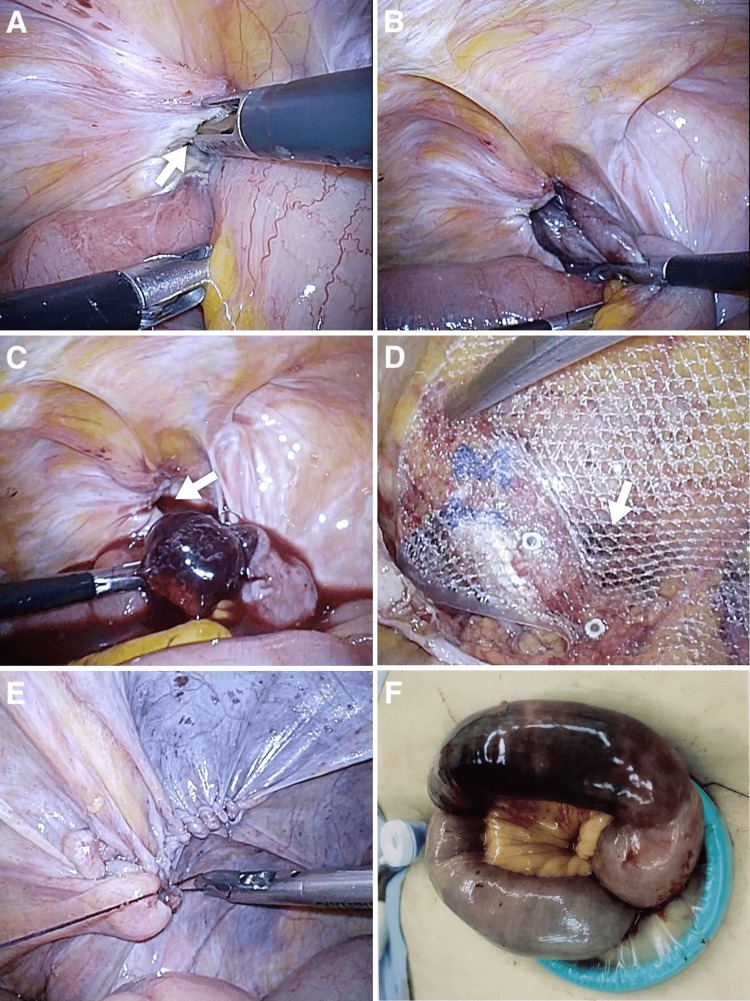
Representative photographs during the operation A. The hernia orifice was enlarged by a peritoneal incision made medially towards the lacunar ligament. The arrow indicates an incision of the peritoneum and part of the lacunar ligament. B. The incarcerated small intestine was reduced by gentle traction with grasping forceps. C. After the strangulated bowel was reduced, bloody ascites was discharged from the hernia sac. The arrow indicates the femoral ring. D. A prosthetic mesh covered the myopectineal orifice, including the hernia orifice. The arrow indicates the femoral ring. E. The peritoneum was closed tightly by a running suture. F. The strangulated bowel was pulled out through the mini-laparotomy incision for resection and anastomosis. Figures  1A-1C, 1D-1E, 1F were photographed in Cases 2, 3, and 1, respectively.

Antibiotic prophylaxis was initiated within one hour of the skin incision and used for one to two days, whereas it was continued for three days when enteric spillage occurred during anastomosis. The outpatient follow-up was scheduled at two weeks, six months, and 12 months after discharge. Hernia recurrence was assessed by clinical examinations through the outpatient department, except for Case 4, where the recurrence was assessed through a telephone inquiry. The surgical wounds were retrospectively classified as clean, clean-contaminated, contaminated, and dirty-infected according to the surgical wound classification of the Centers for Disease Control and Prevention [11].

Case 1

A 64-year-old woman was taken to the emergency department with a chief complaint of abdominal pain that began two hours prior to arrival and was admitted to our hospital. The patient had a past medical history of hypertension and bronchial asthma. Physical examination on the following day revealed a 5-cm mass in the right groin and a somewhat distended abdomen, which was diagnosed as a strangulated femoral hernia by CT scan showing incarcerated small intestine and intestinal obstruction (Figures 2A, 2B). Laboratory evaluation revealed an elevated white blood cell (WBC) count of 13,400 /µL. Emergency surgery was performed on the same day of diagnosis. Laparoscopy revealed that the small intestine was incarcerated in the femoral ring. Unlike the other four cases, an anterior approach, i.e., a femoral approach, was used to reduce the hernia by incising the lacunar ligament. The hernia was classified as primary, F2, and L1 according to the European Hernia Society (EHS) groin hernia classification [12]. The affected portion of bowel was thought to be necrotic because it was a mix of dark red and dark purple on laparoscopic inspection, and a 15-cm segment of small intestine was resected. The operation took 241 minutes, and the blood loss was 28 mL. The patient developed a postoperative seroma, which diminished spontaneously. She was discharged nine days after the operation. Pathological examination revealed ischemic change, epithelial shedding, mucosal and submucosal hemorrhage, and mild infiltration of inflammatory cells. The surgical wound was classified as clean-contaminated. No hernia recurrence was observed at 33 months postoperatively.

**Figure 2:**
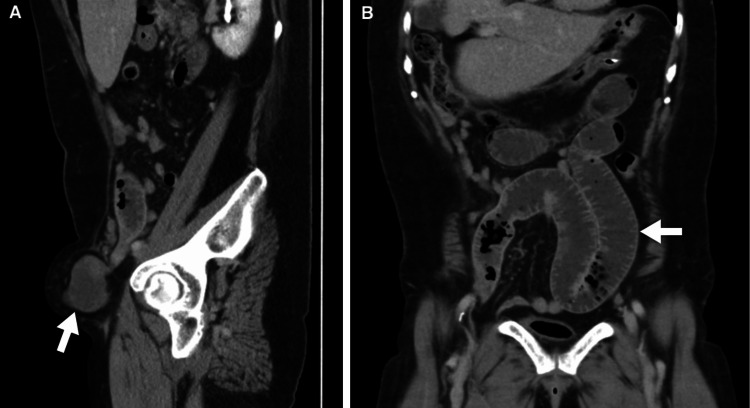
Contrast-enhanced CT scan demonstrating a strangulated femoral hernia and small intestinal obstruction in Case 1 A. The arrow indicates the small intestine protruding in the right groin area (sagittal view). B. The arrow indicates the distended small intestine proximal to the hernia (coronal view).

Case 2

A 90-year-old woman presented to our hospital with two days of periumbilical pain, vomiting, and a 5-cm mass in the right groin. The patient had a history of hypertension, percutaneous coronary intervention for ischemic heart disease, and laparotomy for uterine myoma. Physical examination revealed a right groin mass and a somewhat distended abdomen. A CT scan confirmed the diagnosis of a strangulated femoral hernia involving the small intestine and of intestinal obstruction (Figures 3A, 3B). Laboratory evaluation revealed chronic anemia with hemoglobin (Hb) of 9.8 g/dL, acute kidney injury with blood urea nitrogen (BUN) of 55 mg/dL and serum creatinine (sCr) of 1.7 mg/dL, and elevated inflammation with C-reactive protein (CRP) of 7.9 mg/dL. Emergency surgery was performed as mentioned above. The hernia was classified as primary, F1, and L1 according to the EHS groin hernia classification. The strangulated portion of bowel was dark purple on laparoscopic inspection (Figure 1C), and a 7-cm segment of small intestine was resected. The operation took 159 minutes, and the blood loss was negligible. Cefmetazole was administered for two days. The patient developed postoperative paralytic ileus, which was treated conservatively. She was discharged 15 days after the operation. Pathological examination revealed hemorrhagic mucosal necrosis, transmural hemorrhage, and infiltration of inflammatory cells. The surgical wound was classified as clean-contaminated. No hernia recurrence was observed at 19 months postoperatively.

**Figure 3:**
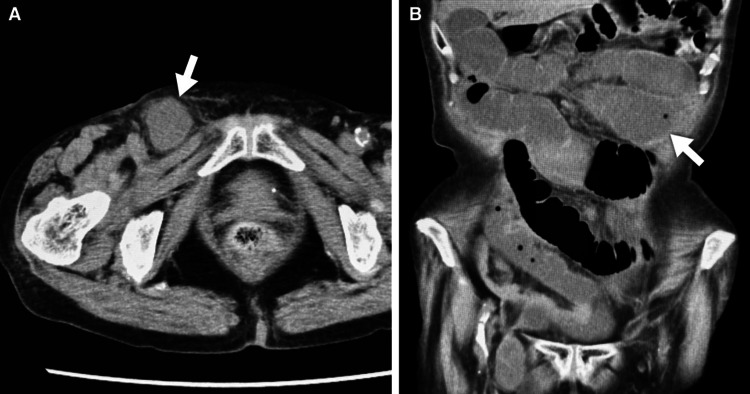
CT scan demonstrating strangulated femoral hernia and small intestinal obstruction in case 2 A. The arrow indicates small intestine protruded in the right groin area (axial view). B. The arrow indicates distended small intestine proximal to the hernia (coronal view).

Case 3

A 95-year-old woman with a history of hypertension and gynecologic surgery was transferred to our hospital with four days of anorexia and vomiting. Physical examination revealed a 6-cm tender mass in the right groin, and a CT scan confirmed the diagnosis of a strangulated femoral hernia and intestinal obstruction (Figures 4A, 4B). Laboratory evaluation revealed a mild anemia with Hb of 10.9 g/dL, chronic kidney disease with sCr of 1.17 mg/dL, and a mild inflammation with CRP of 3.5 mg/dL. Emergency surgery was performed as above. The hernia was classified as primary, F1. The affected portion of bowel was dark purple and dark red, and a 15-cm segment of small bowel was resected. The operation took 108 minutes, and the blood loss was 10 mL. Ceftriaxone was administered for one day. The patient was discharged seven days after the operation without postoperative complications. Pathological examination revealed hemorrhagic necrosis and marked infiltration of inflammatory cells. The surgical wound classification was presumed to be contaminated, considering the necrosis and the marked inflammation. No hernia recurrence was observed at 12 months postoperatively.

**Figure 4:**
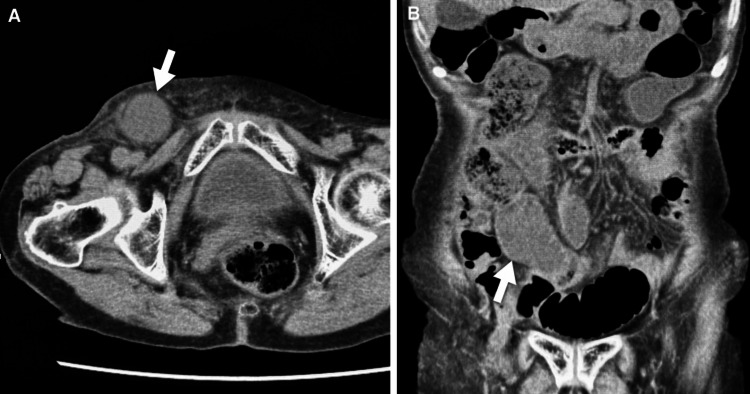
CT scan demonstrating a strangulated femoral hernia and small intestinal obstruction in Case 3 A. The arrow indicates the small intestine protruding in the right groin area (axial view). B. The arrow indicates the distended small intestine proximal to the hernia (coronal view).

Case 4

An 85-year-old woman with a history of hypertension and subarachnoid hemorrhage was transferred to our hospital with right groin pain and nausea that had begun the previous day. Physical examination revealed a 6-cm mass in the right groin and a flat abdomen, and a CT scan confirmed the diagnosis of a strangulated femoral hernia and small intestinal obstruction (Figures 5A, 5B). Laboratory evaluation revealed an elevated WBC count of 14,600 µL and an elevated lactate dehydrogenase of 241 IU/L. Emergency surgery was performed as above. The serosa of the small intestine near the strangulated segment was injured by the tip of the active blade of the SonoSurg® because the SonoSurg® was inserted through the port on the other side of the hernia, and the tip came into contact with the intestine. The hernia was classified as primary, F1. The affected portion of the bowel was dark red and dark purple; a 7-cm segment of small bowel was resected. A small amount of enteric spillage occurred during intestinal anastomosis. The operation took 135 minutes, and the blood loss was 40 mL. Sulbactam/ampicillin was administered for three days. The delirium subsided within a few days, and the patient was discharged eight days after the operation. Pathological examination revealed hemorrhagic mucosal necrosis and transmural hemorrhage. The surgical wound was assumed to be clean-contaminated or contaminated, considering the enteric spillage during the anastomosis. The patient did not visit the outpatient department at six months postoperatively. A recent telephone inquiry revealed that the hernia had not recurred 29 months postoperatively.

**Figure 5:**
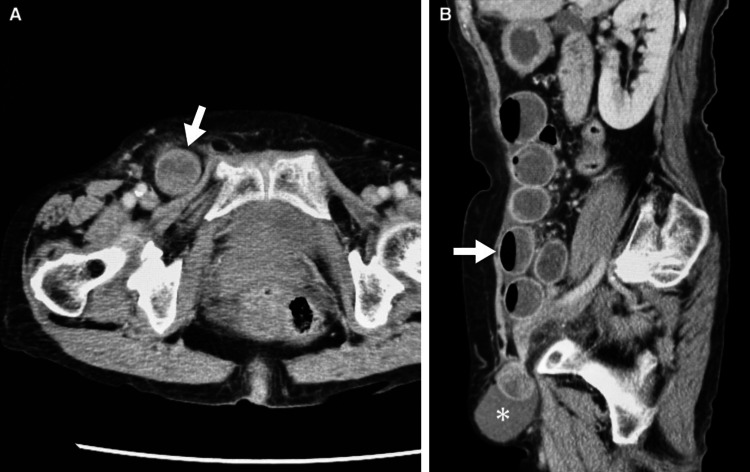
Contrast-enhanced CT scan demonstrating a strangulated femoral hernia and small intestinal obstruction in Case 4 A. The arrow indicates the small intestine protruding in the right groin area (axial view). B. The arrow indicates a distended small intestine proximal to the hernia, and the asterisk indicates fluid in the hernia sac (sagittal view).

Case 5

A 73-year-old man with a history of hypertension, appendectomy, and laparotomy for a perforated gastric ulcer presented to our hospital with three days of anorexia, vomiting, lower abdominal pain, and a 7-cm right groin mass. The patient had also been operated on eight years before for a right inguinal hernia by open herniorrhaphy using the UltraPro hernia system® (Ethicon). The middle and lower abdomen were distended. A CT scan confirmed the diagnosis of a strangulated femoral hernia and extensive small bowel obstruction (Figures 6A, 6B). Laboratory evaluation revealed severe acute kidney injury with a BUN of 60 mg/dL and sCr of 4.8 mg/dL, and elevated inflammation with CRP of 8.8 mg/dL. Surgery was preceded by the placement of a long intestinal tube to relieve bowel distension and the correction of dehydration. The following day, surgery was performed as above except that the Ventralight® ST (Bard) trimmed to 10 x 6 cm was used to cover the femoral ring and fixed by absorbable tacks (SorbaFix™; Bard). The underlay patch of the UltraPro hernia system®, from a previous surgery 8 years ago, had covered the internal inguinal ring and Hesselbach’s triangle. The hernia was classified as recurrent, F1. The serosa of the small intestine near the strangulated segment was lacerated by traction with grasping forceps during hernia reduction. The strangulated portion was dark red and dark purple, and a 10-cm segment of small bowel was resected. Enteric spillage occurred during intestinal anastomosis. The operation took 212 minutes, and the blood loss was 50 mL. Cefmetazole was administered for three days. The patient developed a postoperative groin hematoma, which diminished spontaneously. He was discharged 13 days after the operation. Pathological examination revealed hemorrhagic necrosis and marked infiltration of inflammatory cells. The surgical wound was presumed to be contaminated, considering the necrosis and the marked inflammation. No hernia recurrence was observed at six months postoperatively. However, the recurrence could not be assessed at 12 months because he did not visit the outpatient department on the scheduled day. Also, we could not reach the patient recently.

**Figure 6:**
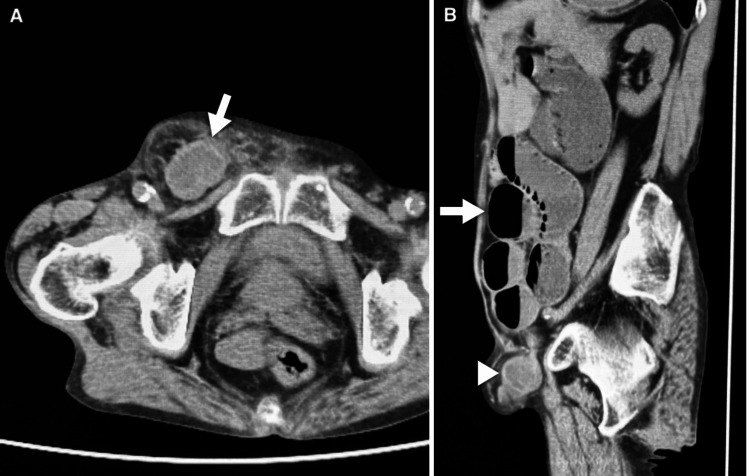
CT scan demonstrating a strangulated femoral hernia and small intestinal obstruction in Case 5 A. The arrow indicates a small intestine protruded in the right groin area (axial view). B. The arrow indicates distended small intestine proximal to the hernia, and the arrowhead indicates the incarcerated intestine (sagittal view).

Table 1 summarizes the clinical characteristics and the postoperative outcomes of the five patients.

**Table 1:** Clinical characteristics and postoperative outcomes of the five patients HT: hypertension; GU: gastric ulcer; EHS: European Hernia Society; USAD: ultrasonically activated device; CMZ: cefmetazole; CTRX: ceftriaxone; SBT/ABPC: sulbactam/ampicillin; CDC: Centers for Disease Control and Prevention

Variables	Case 1	Case 2	Case 3	Case 4	Case 5
Age (years)	64	90	95	85	73
Sex	Female	Female	Female	Female	Male
Onset to surgery (days)	1	2	4	1	4
EHS groin hernia Classification	Primary, F2 and L1	Primary, F1 and L1	Primary, F1	Primary, F1	Recurrent, F1
Mesh	3D Max® Light	3D Max® Light	3D Max® Light	3D Max® Light	Ventralight® ST
Tissue injury during reduction	No	No	No	Serosal burn by USAD	Serosal laceration by traction
Conversion to open surgery	No	No	No	No	No
Bowel resection length (cm)	15	7	15	7	10
Operation Time (min)	241	159	108	135	212
Blood loss (mL)	28	negligible	10	40	50
Perioperative antibiotic use	CMZ for 2 days	CMZ for 2 days	CTRX for 1 day	SBT/ABPC for 3 days	CMZ for 3 days
Postoperative hospital stay (days)	9	15	7	8	13
Postoperative complication	Groin seroma	Paralytic ileus	No	Delirium	Groin hematoma
Pathological findings of the small intestine	Ischemic change	Mucosal necrosis	Necrosis with marked inflammation	Mucosal necrosis	Necrosis with marked inflammation
CDC Surgical wound classification	Clean-contaminated	Clean-contaminated	Contaminated	Clean-contaminated or contaminated	Contaminated
Observation period (months)	33	19	12	29	6
Hernia recurrence	No	No	No	No	No
Method to assess recurrence	Clinical exam	Clinical exam	Clinical exam	Telephone inquiry	Clinical exam

## Discussion

In this case series, hernia reduction was performed laparoscopically in four cases. Although the small intestine was injured during hernia reduction in two cases, there was no perforation or the resulting contamination of the surgical field. In addition, none of the cases needed conversions to the open anterior approach or laparotomy. These results suggest that TAPP repair with concomitant bowel resection for a strangulated femoral hernia is feasible. Moreover, no patients had infectious complications, leading us to surmise that this procedure can be performed safely with a low incidence of mesh infection.

Laparoscopic surgery is not consistently considered to be suitable for strangulated groin hernia, possibly due in part to the technical difficulties in reducing the strangulated hernia contents and the risk for iatrogenic injuries. In this case series, strangulated hernias were reduced laparoscopically with a medial incision of the peritoneum and part of the lacunar ligament and traction on the herniated intestine, except in the first case, where the hernia was reduced from the anterior approach. The tissue pad was inserted between the peritoneum to be incised and the small intestine to avoid injury by the active blade. In Case 4, a serosal burn was caused by the tip of the active blade inserted through the port on the other side of the hernia, indicating that the device should be inserted through the port on the same side as the hernia to avoid contact of the active blade. In Case 5, a serosal laceration appeared to result from traction on the intestine under inadequate enlargement of the hernia orifice, suggesting that the hernia orifice should be enlarged enough to allow easy reduction. Several studies have reported the laparoscopic techniques to enlarge the hernia orifice prior to femoral or inguinal hernia reduction. Rebuffat et al. reported that the hernia ring could be enlarged through a ventromedial incision in the cases of femoral hernias and direct inguinal hernias and through a ventrolateral incision in the cases of indirect inguinal hernias to prevent vessel injury [7]. Matsuda et al. attempted an initial incision and enlargement of the hernia orifice before retraction of incarcerated tissue in laparoscopic surgery for strangulated inguinal and femoral hernias and found that no tissue injuries were encountered with the "orifice first approach". An incision was made at the 12 o’clock position (in a ventral direction) using an ultrasonically activated device [8]. Furthermore, Liu et al. pointed out that hernia ring incision was a good means of hernia reduction in laparoscopic surgery for strangulated inguinal hernias, resulting in an easy reduction of hernia contents without additional tissue injuries. The hernia orifice was enlarged ventromedially for direct hernias and ventrolaterally for indirect hernias to avoid injury of the inferior epigastric artery. They used an electronic hook to incise the hernia ring in the preperitoneal plane so that the peritoneum could provide protection to the incarcerated bowel from iatrogenic injuries [13]. Thus, the incision and enlargement of the hernia orifice can facilitate the laparoscopic reduction of strangulated hernia contents. The hernia orifice should be incised with caution to avoid contact of the active blade with the incarcerated bowel and should be adequately enlarged to avoid excess traction on the bowel.

The feasibility of laparoscopic surgery for a strangulated groin hernia can be evaluated by the ability to reduce hernia content and the rate of conversion to open surgery. In this case series, strangulated hernias were reduced laparoscopically as mentioned above, and none of the cases needed conversion to open surgery. The results of our report are in keeping with other studies supporting the view that laparoscopic surgery for strangulated groin hernia is feasible [2,6,13]. Part of our criteria for patient selection were comparable to the criteria for patient selection in laparoscopic surgery for strangulated groin hernia in the literature [14,15]. As regards bowel obstruction, all patients in our case series had a mild to moderate bowel distension at the time of surgery, although the patient in Case 5 had a distended abdomen with a severe bowel distension before the placement of a long intestinal tube. Given that bowel distension due to intestinal obstruction can lead to conversion to open surgery [7], patients would need to be selected according to the extent of abdominal distension in order to perform and complete the laparoscopic procedure for a strangulated groin hernia as planned.

None of our cases had mesh infection after TAPP with concomitant bowel resection, which is in line with earlier reports. Smith et al. reported a case series of nine patients with strangulated groin hernias operated on by TAPP repair with concomitant bowel resection with no mesh infection [16]. In cohort studies, Rebuffat et al. reported 28 irreducible strangulated hernias repaired by TAPP, which included eight cases of concomitant bowel resection with no infectious complications [7]. Similarly, Matsuda et al. reported 11 cases of strangulated inguinal hernia treated by TAPP, out of which intestinal resection had been required in five cases with no mesh infection [8]. In addition, Liu et al. reported 45 strangulated groin hernias treated by TAPP, including five cases requiring bowel resection with no mesh infection [9]. More recently, Correia de Sá et al have reported, in a meta-analysis including the studies by Rebuffat et al. [7], Matsuda et al. [8], and Liu et al. [9], as well as five other cohort studies of TAPP repair for strangulated groin hernia, that TAPP repair with visceral resection has been performed in 25 patients with no complications related to the mesh [15]. Matsuda et al. surmised that the closure of the peritoneum at the completion of TAPP and the extracorporeal intestinal resection separated the surgical field from the preperitoneal inguinal space and allowed for prosthesis usage with minimal bacterial contamination [8]. In light of this viewpoint, we conjecture that the separation between the peritoneal cavity and the mesh by a tight running suture of peritoneum without gaps at the end of TAPP repair is essential to avoid the contact of the anastomotic site and minimize the risk of mesh infection. Therefore, the low probability of mesh infection in TAPP repair and concomitant bowel resection for strangulated groin hernia may be partly attributable to the avoidance of contact between the anastomotic site and the prosthetic mesh.

It is suggested not to use mesh during emergency groin hernia repair in contaminated or dirty/infected wounds [17]. Initially, we had considered surgical wounds involving necrotic bowel resection to be clean-contaminated. Although the CDC surgical wound classification does not refer to necrotic bowel, the American College of Surgeons-defined surgical wound classification includes incisions in which necrotic tissue is encountered without evidence of purulent drainage as contaminated wounds [11,18]. Classification of the surgical wounds in the five cases was performed based on the results of pathological examination. The surgical wounds were regarded as clean-contaminated when the necrosis of the intestine was confined to the mucosa because it seemed unlikely that bacteria could spread across the viable tissue in the outer layer of the intestine. On the other hand, the surgical wounds were regarded as contaminated when the necrosis was not confined to the mucosa and a marked inflammatory infiltrate was present. The extent of ischemia/strangulation can vary from just a compromised state to a truly non-vital state, which may lead to different risks of contamination. Therefore, evaluation of contamination due to necrosis on laparoscopic inspection may be needed to select the patients and perform this procedure more safely. In addition, enteric spillage potentially contaminating the surgical fields was taken into account in the wound classification of Cases 4 and 5 [11], although the surgical field of mesh placement was different from where anastomosis was performed. However, caution needs to be exercised during anastomosis to avoid a gross enteric spillage, causing contaminated wounds, and to minimize the risk of infection.

One limitation of this report is the small number of cases. Secondly, no comparison of results with other surgical methods has been made. For example, if results were compared with the already recommended, concomitant bowel resection and hernia repair with a prosthetic mesh through an open anterior approach [10], it could be evaluated whether TAPP with concomitant bowel resection is a safe and preferred surgical procedure.

## Conclusions

Although our report includes a limited number of cases, taken together with earlier reports, TAPP repair with concomitant bowel resection appears feasible in selected patients for strangulated groin hernia. Attention needs to be paid to the techniques in reducing strangulated hernias, such as the use of dissecting devices and the adequate enlargement of the hernia orifice to avoid tissue injury. In addition, evaluation of contamination of the strangulated bowel, a complete closure of the peritoneum after TAPP, and avoidance of enteric spillage during anastomosis may minimize the risk of mesh complications. Further accumulation of cases and prospective comparative studies is warranted to verify that this surgical procedure can be performed safely with a low incidence of infectious complications for the treatment of strangulated groin hernia.
